# Resveratrol Counteracts Inflammation in Human M1 and M2 Macrophages upon Challenge with 7-Oxo-Cholesterol: Potential Therapeutic Implications in Atherosclerosis

**DOI:** 10.1155/2014/257543

**Published:** 2014-05-08

**Authors:** Brigitta Buttari, Elisabetta Profumo, Luca Segoni, Daniela D'Arcangelo, Stefania Rossi, Francesco Facchiano, Luciano Saso, Rita Businaro, Luigi Iuliano, Rachele Riganò

**Affiliations:** ^1^Department of Infectious, Parasitic and Immune-Mediated Diseases, Istituto Superiore di Sanità, Viale Regina Elena 299, 00161 Rome, Italy; ^2^Laboratory of Vascular Pathology, Istituto Dermopatico dell'Immacolata, IDI-IRCCS, 00167 Rome, Italy; ^3^Department of Hematology, Oncology and Molecular Medicine, Istituto Superiore di Sanità, 00161 Rome, Italy; ^4^Department of Physiology and Pharmacology “Vittorio Erspamer”, La Sapienza University of Rome, 00181 Rome, Italy; ^5^Department of Medico-Surgical Sciences and Biotechnology, Sapienza University of Rome, 04100 Latin, Italy

## Abstract

Macrophages consist of two main subsets: the proinflammatory M1 subset and the anti-inflammatory M2 one. 7-oxo-cholesterol, the most abundant cholesterol autoxidation product within atherosclerotic plaque, is able to skew the M1/M2 balance towards a proinflammatory profile. In the present study, we explored the ability of the polyphenolic compound resveratrol to counteract the 7-oxo-cholesterol-triggered proinflammatory signaling in macrophages. Resveratrol-pretreated human monocyte-derived M1 and M2 macrophages were challenged with 7-oxo-cholesterol and analyzed for phenotype and endocytic ability by flow cytometry, for metalloproteinase- (MMP-) 2 and MMP-9 by gelatin zymography, and for cytokine, chemokine, and growth factor secretome by a multiplex immunoassay. We also investigated the NF-**κ**B signaling pathway. In the M1 subset, resveratrol prevented the downregulation of CD16 and the upregulation of MMP-2 in response to 7-oxo-cholesterol, whereas in M2 macrophages it prevented the upregulation of CD14, MMP-2, and MMP-9 and the downregulation of endocytosis. Resveratrol prevented the upregulation of several proinflammatory and proangiogenic molecules in both subsets. We identified modulation of NF-**κ**B as a potential mechanism implicated in 7-oxo-cholesterol and resveratrol effects. Our results strengthen previous findings on the immunomodulatory ability of resveratrol and highlight its role as potential therapeutic or preventive compound, to counteract the proatherogenic oxysterol signaling within atherosclerotic plaque.

## 1. Introduction

Atherosclerosis is a chronic inflammatory disease characterized by accumulation of immune cells within the atherosclerotic plaque [1, 2], including macrophages that are the main cellular components [3]. Human atherosclerotic plaque is composed of a large mixture of elements, predominantly lipids and oxidized lipids, lipid-loaded macrophages, and smooth muscle cells, forming foam cells. Plaque contents undergo dynamic changes during the plaque's progression, being in a constant interaction with the circulating blood [4]. The fate of atherosclerotic plaques is highly dependent upon the balance between recruitment and activation of monocyte-derived macrophages, upon their clearance from the vessel wall [5] and upon macrophage polarization state [6]. Macrophage M1 and M2 activation phenotypes represent two ends of a functional spectrum of macrophage polarization state [6], which may accelerate or decelerate atherosclerotic disease progression through igniting or cooling down inflammatory reactions. The proinflammatory M1, or classically activated subset, produces inflammatory cytokines and is a leading source of reactive oxygen species in atherosclerotic lesions [7, 8]. M2, or alternatively polarized macrophages, are a heterogeneous group of cells that show an anti-inflammatory phenotype and appear to be critical for the resolution of inflammation [7, 9]. Plasticity is a hallmark of cells of the monocyte-macrophage lineage [10]. The molecules and mechanisms associated with plasticity and polarized activation of macrophages may provide a basis for innovative diagnostic and therapeutic approaches [10]. We have recently demonstrated that 7-oxo-cholesterol, the most abundant cholesterol autoxidation product within atherosclerosis lesions [11–14], is able to affect human macrophage polarization by skewing the M1/M2 balance towards a proinflammatory profile [15]. Because proinflammatory macrophages play a key role in atherogenesis, plaque rupture, and subsequent clinical events, the inhibition of this new 7-oxo-cholesterol-triggered proinflammatory pathway by the use of a therapeutic approach capable to modulate the M1/M2 macrophage balance within atherosclerotic plaque might provide interesting therapeutic prospects in reducing atherosclerosis and/or in the prevention of plaque rupture. There is emerging evidence that beside current Western therapies, many alternative and nutrition therapies have the ability to modulate the immune system and disrupt the proinflammatory cascade through a variety of mechanisms, including antioxidant effects, alterations in cell signaling, cytokines, and proinflammatory mediators [16]. Resveratrol, a polyphenolic compound found in red wine and grapes, plays a potentially important role in many disorders [17]. It possesses antioxidant, anti-inflammatory, antiproliferative, and antiangiogenic effects and many signaling pathways are among its molecular targets.

With regard to anti-inflammatory and immunomodulatory effects, the process activated by resveratrol has not been clearly established, even though it does not seem to be mechanically as simple as a nonspecific inhibition of inflammation [16].

In this study, we explored the ability of resveratrol to counteract the proinflammatory signaling triggered by 7-oxo-cholesterol in M1 and M2 macrophage subsets and we investigated a potential mechanism implicated in such prevention. By the use of flow cytometry, gelatin zymography, and a multiplex immunoassay we demonstrated that resveratrol was able to counteract oxysterol-induced proinflammatory phenotypical and functional changes in both M1 and M2 subsets.

## 2. Materials and Methods

### 2.1. Reagents

Recombinant human (rh) granulocyte-macrophage colony-stimulating factor (GM-CSF) and rh macrophage colony-stimulating factor (M-CSF) were from R&D System (Minneapolis, MN). Foetal bovine serum (FBS) was from Hyclone Laboratories (Logan, UT). Anti-CD14-coated microbeads were from Miltenyi Biotec (Gladbach, Germany). RPMI 1640 was from GIBCO (Paisley, UK). Phycoerythrin- (PE-) conjugated monoclonal antibodies (mAbs) to CD1a and human leukocyte antigen-D region-related (HLA-DR) and fluorescein isothiocyanate- (FITC-) conjugated mAbs to CD16 were from PharMingen (San Diego, CA); allophycocyanin- (APC-) conjugated mAbs to CD14 and CD163 (clone GHI/61) were from BioLegend (San Diego, CA). 7-oxo-cholesterol, resveratrol, and the other chemicals were from Sigma-Aldrich (Milan, Italy). Resveratrol was dissolved in ethanol at 50 mg/mL and aliquots were frozen at −80°C under sterile conditions.

### 2.2. Preparation of Human Monocyte-Derived M1 and M2 Macrophages

Blood samples from 4 healthy blood donors from the Transfusion Center at the Sapienza University of Rome were used to obtain peripheral blood mononuclear cells (PBMCs). The study was conducted in accordance with the Helsinki Declaration of 1975 and 1983.

Monocytes were obtained from PBMCs, as described previously [18]. In brief, PBMCs were isolated by density gradient (Lympholyte, Cedarlane, Oxford, UK). CD14+ monocytes were purified by incubating PBMCs with anti-CD14-coated microbeads, followed by sorting with a magnetic device (MiniMacs, Miltenyi Biotec). Monocytes were induced to differentiate for 6 days in cell culture dishes (100 mm) (BD-Biosciences, San Diego, CA), in the presence of either rhGM-CSF (10 ng/mL) to obtain M1 macrophages or rhM-CSF (10 ng/mL) to obtain M2 ones. Cells were cultured at 8 × 10^5^ cells/mL in RPMI 1640—supplemented with 1% nonessential amino acids, 1% sodium pyruvate, 50 U/mL penicillin, 50 *μ*g/mL streptomycin, 5 × 10^−5 ^M 2-mercaptoethanol, and 10% FBS.

### 2.3. Flow Cytometric Analysis of Monocyte-Derived M1 and M2 Macrophage Phenotype

Flow cytometric analysis was performed before any treatment to confirm M1 and M2 induction and was later used to evaluate the effect of resveratrol and 7-oxo-cholesterol on macrophage phenotype. The presence of characteristic phenotypic surface markers (CD14^high^, CD16^high^, CD163^low^, and HLA-DR^high^ for M1 and CD14^high^, CD16^low^, CD163^high^, and HLA-DR^low^ for M2) was analyzed on a FACSCanto and using CellDIVA software (BD-Biosciences). Macrophages were stained with PE-conjugated mAb to HLA-DR, FITC-conjugated mAb to CD16, APC-conjugated mAbs to CD14 and CD163 (clone GHI/61) or with isotype-matched control mAbs for 30 minutes at 4°C. All samples were analyzed by flow cytometry (FACSCanto, BD-Biosciences).

### 2.4. Treatment of M1 and M2 Macrophages with Resveratrol and Exposure to 7-Oxo-Cholesterol

On day 6, adherent macrophages were collected and 7 × 10^5^ cells were cultured in 24 well plates (BD-Biosciences) and treated or not with resveratrol (30 *μ*M) for 1 hour at 37°C, 5% CO_2_. Resveratrol concentration was chosen on the basis of preliminary dose/response experiments using concentrations ranging from 3 to 80 *μ*M. Then cells were stimulated with 7-oxo-cholesterol in ethanol (15 *μ*M) for 20 hours. LPS-treated cells (100 ng/mL) were used as positive control. Macrophages pretreated or not with resveratrol and stimulated with 7-oxo-cholesterol were exposed to 0.2% trypan blue and then counted in a hemocytometer to calculate cell viability and the percentage of dead cells.

### 2.5. Flow Cytometric Analysis of Macrophage Endocytosis

To deliver more information on M1/M2 macrophage discrimination, we investigated macrophage mannose receptor-mediated endocytosis as previously described [15]. In brief, macrophages treated or not with resveratrol for 1 hour at 37°C and then stimulated with 7-oxo-cholesterol (15 *μ*M) at 37°C for 20 hours were incubated (2 × 10^5^ cells/sample) with FITC-dextran (1 mg/mL; molecular mass 40.000, Sigma) for 30 min at 37°C. After incubation, macrophages were washed twice with PBS and fixed with 1% formaldehyde. At least 5 × 10^3^ cells/sample were analyzed by flow cytometry (FACSCanto, BD-Biosciences).

### 2.6. Assessment of MMP-2 and MMP-9 by Gelatin Zymography

The effect of resveratrol and 7-oxo-cholesterol treatment on macrophage function was evaluated by determining metalloproteinases activity. Macrophage culture supernatants were collected after pretreatment with resveratrol and stimulation with 7-oxo-cholesterol (15 *μ*M). MMP-2 and MMP-9 activity was measured by gelatin zymography as described previously [15]. Cell supernatants were subjected to polyacrylamide gel electrophoresis (SDS-PAGE). Gels (10.5%) were copolymerised with gelatin (0.9%). For each sample, 6 *μ*L of cell supernatant in 6 *μ*L of loading buffer (Bio-Rad) was loaded under native conditions. Electrophoresis was carried out using the mini-gel slab apparatus Mini Protean 3 (Bio-Rad, Milan, Italy) at a constant voltage of 150 V. Following electrophoresis, gels were washed in renaturating buffer (2.5% Triton X-100 in 50 mM Tris-HCl, pH 7.5) for 1 h in an orbital shaker. Then, the zymograms were incubated for 18 h at 37°C in Tris buffer pH 7.5 (0.15 M NaCl, 10 mM CaCl_2_, 0.02% NaN_3_ in 50 mM Tris-HCl). Gels were then stained with Coomassie blue and destained with 7% methanol and 5% acetic acid. Areas of enzymatic activity, which appeared as clear bands over the dark background, were quantified using ChemiDoc densitometer (Bio-Rad, Hercules, CA). For analysis purpose, the image was digitally inverted so that the integration of bands was reported as positive values. The pixel density was determined after background subtraction and used to calculate the integrated density of a selected band that was reported as the mean of three different measurements of the same gel for each sample run in triplicate.

### 2.7. Assessment of MMP-2 and MMP-9 by Western Blotting

The identification of macrophage-derived MMP-2 and -9 was performed by Western blotting. Supernatants were subjected to 10.5% SDS-PAGE and then blotted onto polyvinylidene fluoride membranes (Immobilon-P, Millipore, Tullagreen, Ireland). Blots were incubated with anti-human MMP-2 or -9 Abs (Santa Cruz Biotechnology, Inc., Santa Cruz, CA) and then with anti-goat HRP-coupled secondary Ab (Bio-Rad, Hercules, CA). Immunoreactivity was assessed by the chemiluminescence reaction with the ECL system (Amersham, Buckinghamshire, UK) and analyzed by ChemiDoc densitometer (Bio-Rad).

### 2.8. Secretome Profile of Cytokines, Chemokines, and Growth Factors in Macrophage Culture Supernatants

Conditioned media were harvested and processed for cytokine analysis in duplicate with a custom Bio-Rad Bio-Plex human cytokine reagent kit for IL-1 receptor antagonist (IL-1ra), IL-6, IL-8, IL-10, IL-12, granulocyte colony stimulating factor (G-CSF), GM-CSF, interferon-inducible protein (IP-10), monocyte chemoattractant protein-1 (MCP-1), macrophage inflammatory protein 1-*α* (MIP-1*α* or CCL3), MIP-1*β* (CCL4), regulated and normal T cell expressed and secreted (RANTES), TNF-*α*, and vascular endothelial growth factor (VEGF) according to the manufacturer's instructions (Bio-Rad, Hercules, CA). Data were acquired on the Bio-Rad Bio-Plex 200 reader equipped with a magnetic workstation and analyzed using Bio-Plex software version 6.0 (Bio-Rad). Values presenting a coefficient of variation beyond 10% were discarded before the final data analysis. Minimum levels of detection (pg/mL) were 4.89 for IL-1ra, 0.23 for IL-6, 0.58 for IL-8, 0.17 for IL-10, 0.26 for IL-12, 0.1 for G-CSF, 2.26 for GM-CSF, 1.83 for IP-10, 3.56 for MCP-1, 2.38 for MIP-1*α*, 2.69 for MIP-1*β*, 0.49 for RANTES, 8.84 for TNF-*α*, 3.54 for VEGF.

### 2.9. Nuclear Factor-*κ*B (NF-*κ*B) Translocation

The NF-*κ*B (p65 and p50) transcription factor assay kit (Active Motive Carlsbad, CA, USA) was used to monitor NF-*κ*B activation as previously described [19]. Macrophages treated or not with resveratrol for 30 min at 37°C and then stimulated with 7-oxo-cholesterol (15 *μ*M) at 37°C for 1 hour were lysed. Protein content was quantified, and activated levels of p65 and p50 subunits were determined in equal amounts of lysates by the use of Abs directed against the subunits bound to the oligonucleotide containing the NF-*κ*B consensus binding site.

### 2.10. Statistical Analysis

Mean values and standard deviations were calculated for each variable under study. All the statistical procedures were performed by GraphPad Prism software (San Diego, CA, USA). Data were tested for Gaussian distribution with the Kolmogorov-Smirnov test. Normally distributed data were analysed using one-way ANOVA with a Bonferroni* post hoc* test to evaluate the statistical significance of intergroup differences in all the tested variables. *P* values <0.05 were considered statistically significant.

## 3. Results

### 3.1. Resveratrol Prevents 7-Oxo-Cholesterol-Induced CD16 and CD14 Changes in M1 and M2 Macrophage Subsets

**Figure 1:**
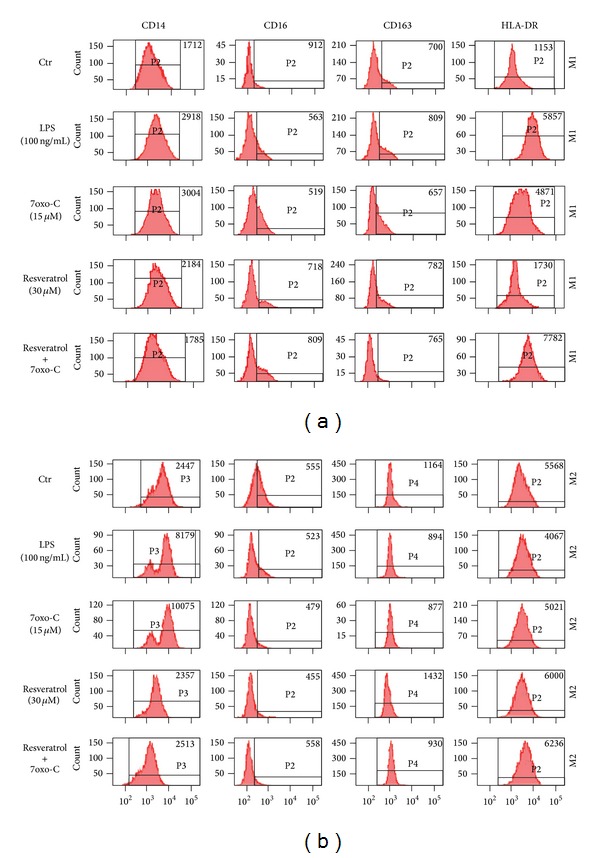
Flow cytometric analysis of differentiation and activation surface markers on M1 and M2 macrophage subsets. Resveratrol prevented 7-oxo-cholesterol (7oxo-C) induced CD16 and CD14 changes in M1 (a) and M2 (b) macrophage subsets. Polarized M1 and M2 macrophages pretreated or not with resveratrol for 1 hour were stimulated with 7oxo-C (15 *μ*M) for 20 hours and then analyzed for surface molecule expression by flow cytometry. Macrophages stimulated with LPS (100 ng/mL) were used as positive control. The results of one representative experiment of three are shown. The number in the histograms shows the mean fluorescence intensity.

The impact of resveratrol on the 7-oxo-cholesterol-induced phenotypical changes in M1 and M2 macrophages was assessed by flow cytometric analysis of the differentiation and activation surface markers CD14, CD16, CD163, and HLA-DR (Figure 1, Table 1). A reduction in CD16 expression (*P* < 0.001) and an increase in HLA-DR expression (*P* < 0.05) were observed on the M1 subset, whilst M2 subset showed increased CD14 expression (*P* < 0.001). Treatment of cells with resveratrol before challenge with oxysterol prevented CD16 downregulation in M1 and CD14 upregulation in M2 macrophages (7-oxo-cholesterol plus resveratrol versus 7-oxo-cholesterol: CD16, *P* < 0.01; CD14, *P* < 0.001). Resveratrol* per se* did not cause any surface marker changes.

### 3.2. Resveratrol Prevents the Impairment of Endocytosis in M2 Macrophages in Response to 7-Oxo-Cholesterol

**Figure 2:**
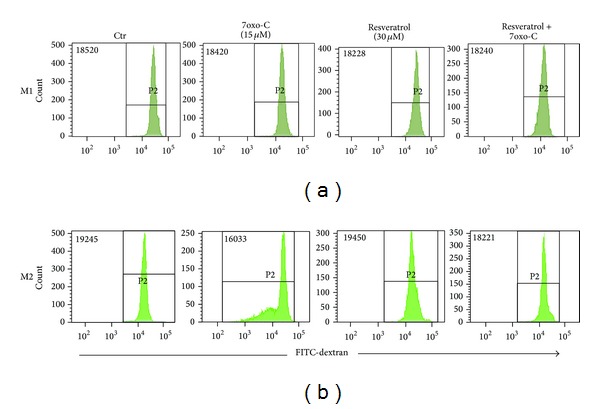
Analysis of macrophage endocytosis. Resveratrol prevented the impairment of endocytosis in M2 macrophages in response to 7-oxo-cholesterol (7oxo-C). M1 (a) and M2 (b) macrophages—pretreated or not with resveratrol (30 *μ*M) for 1 hour and then incubated with 7oxo-C (15 *μ*M) for 20 h or left unstimulated—were added with FITC-dextran (1 mg/mL) and incubated for 30 minutes at 37°C at 5% CO_2_. The cellular uptake was analyzed by flow cytometry. The results of one representative experiment of three are shown. The number in the histograms shows the mean fluorescence intensity.

Flow cytometric analysis showed that resveratrol pretreatment prevented the reduction of M2 macrophage ability to take up FITC-dextran in response to 7-oxo-cholesterol whereas it had no effect on 7-oxo-cholesterol-treated M1 macrophage endocytosis (Figure 2). Resveratrol* per se* did not change the endocytic ability of unstimulated M1 and M2 macrophages.

### 3.3. Resveratrol Prevents 7-Oxo-Cholesterol-Induced MMP-2 and MMP-9 Production in M1 and M2 Macrophage Subsets

**Figure 3:**
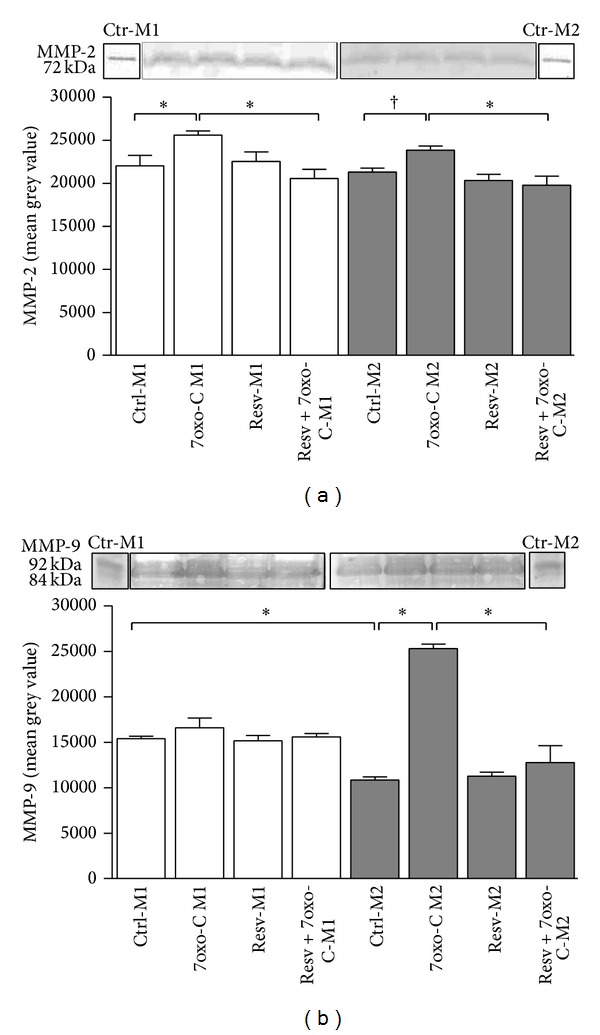
Gel zymography for MMP-2 and MMP-9 detection. Pretreatment of cells with resveratrol prevented upregulation of MMP-2 (a) in M1 and M2 subsets and of MMP-9 (b) in M2 macrophages in response to 7-oxo-cholesterol (7oxo-C). Culture supernatants of polarized M1 (□) and M2 (■) macrophages treated or not with resveratrol (Resv; 30 *μ*M) for 1 hour and then stimulated with 15 *μ*M 7oxo-C for 20 hours or left unstimulated were subjected to acrylamide gel electrophoresis and the gelatinolytic activity was determined by classical zymography as described in Section 2. Results are expressed as means ± SD of four independent experiments (*P < 0.001; ^†^ *P* < 0.05). Representative Western blotting and zymograms are reported on the top of the bar plot.

The impact of resveratrol on macrophage functions was investigated by determining its ability to modulate the MMP-2 and MMP-9 production in response to 7-oxo-cholesterol. Analysis of zymograms for proteolytic activity of macrophage supernatants demonstrated that M1 and M2 macrophages constitutively express the pro-MMP-2 (72 kDa) and pro- and active forms of MMP-9 (92 and 84 kDa). 7-oxo-cholesterol upregulated the expression of MMP-2 in M1 and M2 subsets and of MMP-9 in the M2 subset (Figure 3). Pretreatment of cells with resveratrol prevented upregulation of MMP-2 in M1 and M2 subsets and of MMP-9 in M2 macrophages in response to 7-oxo-cholesterol (*P* < 0.001) (Figure 3). Resveratrol* per se* did not cause any change in metalloproteinase expression. Western blotting showed that the 72 kDa and the 92–84 kDa gelatinolytic activities observed in the zymograms corresponded to MMP-2 and MMP-9, respectively.

### 3.4. Resveratrol Prevents 7-Oxo-Cholesterol-Induced Proinflammatory and Proangiogenic Molecule Production by M1 and M2 Macrophage Subsets

**Figure 4:**
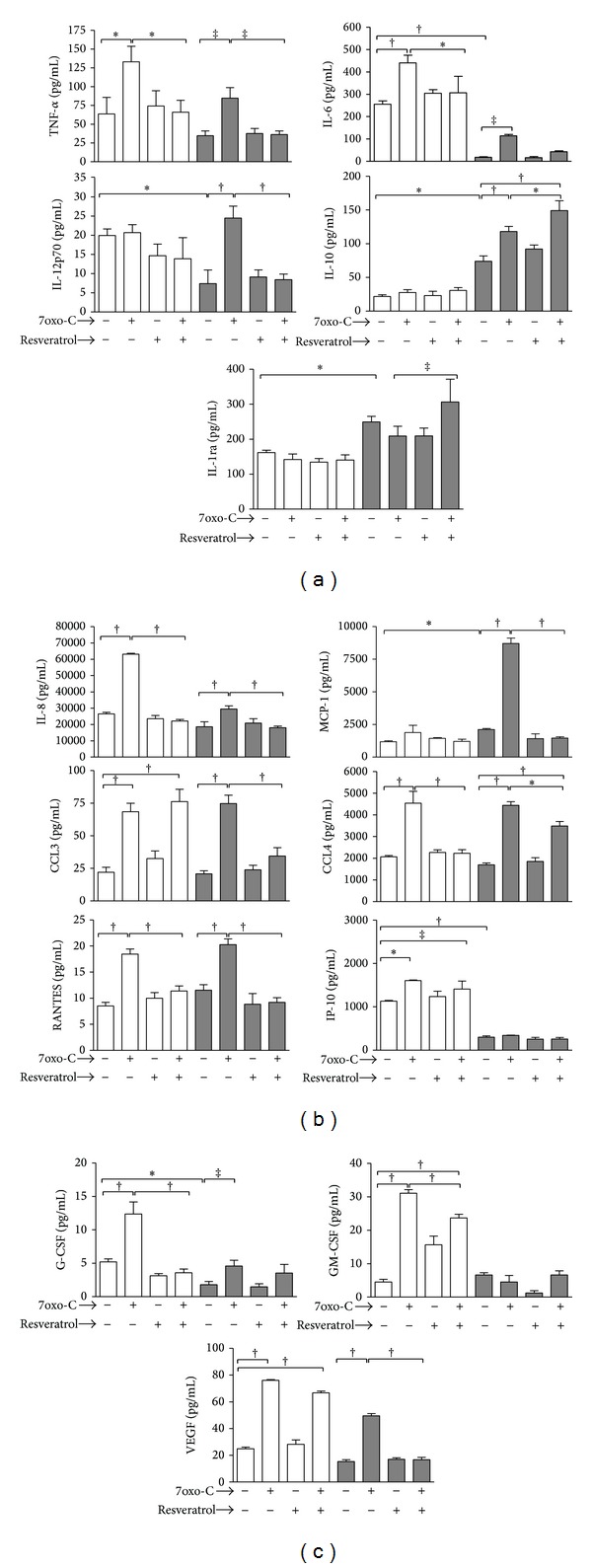
Secretome profile of cytokines, chemokines, and growth factors in M1 and M2 macrophages pretreated or not with resveratrol before stimulation with 7-oxo-cholesterol. Polarized M1 (□) and M2 (■) macrophages were stimulated with 15 *μ*M 7-oxo-cholesterol (7oxo-C) for 20 hours after pretreatment or not with resveratrol (30 *μ*M) for 1 hour at 37°C, 5% CO_2_. At the end of incubation time, supernatants were analyzed for cytokines (a), chemokines (b), and growth factors (c) release using a commercially available multiplex bead-based sandwich immunoassay kit, as described in Section 2. Results are expressed as means ± SD of three independent experiments (*P < 0.01; ^†^ *P* < 0.001; ^‡^ *P* < 0.05).

To investigate the impact of resveratrol on proinflammatory macrophage activation in response to 7-oxo-cholesterol, we screened the secretome profile for cytokines, chemokines, and growth factors released in the culture supernatants by M1 and M2 macrophages treated or not with resveratrol before stimulation with the oxysterol (Figure 3). 7-oxo-cholesterol potentiated the proinflammatory capacity of M1 cells by triggering statistically significant upregulation of the cytokines TNF-*α* and IL-6 (Figure 4(a)), of the chemokines IL-8, CCL3, CCL4, RANTES, and IP-10 (Figure 4(b)), and of the growth factors G-CSF, GM-CSF, and VEGF (Figure 4(c)). It also skewed M2 cell polarization towards a M1-like phenotype by increasing the production of the cytokines TNF-*α*, IL-6, and particularly of IL-12, of the chemokines IL-8, MCP-1, CCL3, CCL4, and RANTES, and finally the production of the growth factors G-CSF and VEGF. It also increased the production of the anti-inflammatory cytokine IL-10.

In M1 macrophages, resveratrol pretreatment significantly prevented TNF-*α* and IL-6 upregulation observed in response to 7-oxo-cholesterol (*P* < 0.01). It also prevented the upregulation of the chemokines IL-8, CCL-4, and RANTES and of the growth factors G-CSF and GM-CSF (*P* < 0.001).

In the M2 macrophage subset, resveratrol pretreatment significantly prevented TNF-*α* (*P* < 0.05) and IL-12 (*P* < 0.001) upregulation in response to 7-oxo-cholesterol and increased IL-10 (*P* < 0.01) and IL-1ra production (*P* < 0.05). It also prevented IL-8, MCP-1, CCL3 (*P* < 0.001), CCL-4 (*P* < 0.01), RANTES, and VEGF upregulation (*P* < 0.001). Resveratrol* per se* did not cause any change in the secretome profile.

### 3.5. Resveratrol Prevents NF-*κ*B Activation in Response to 7-Oxo-Cholesterol

**Figure 5:**
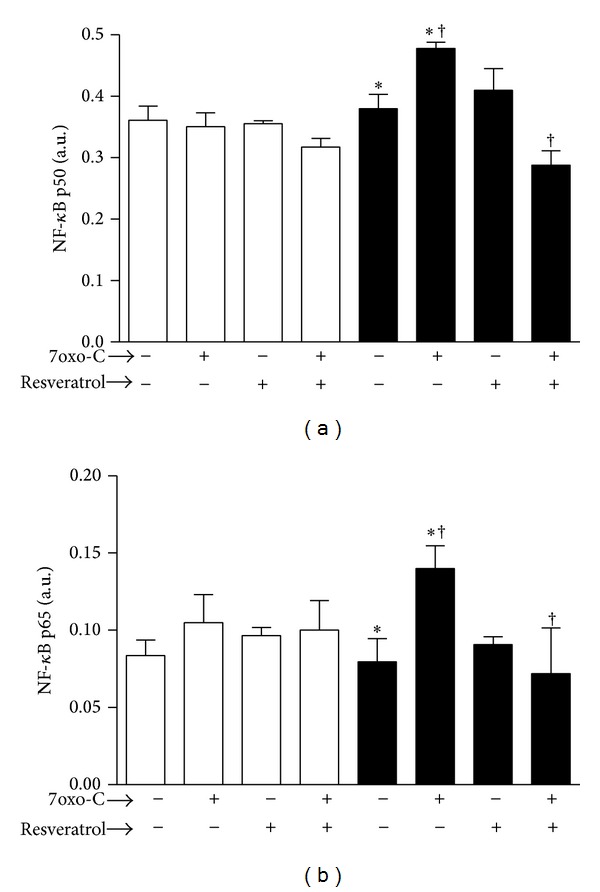
NF-*κ*B activation in M1 and M2 macrophages. 7-oxo-cholesterol (7oxo-C) stimulation significantly increased active NF-*κ*B p50 (a) and p65 (b) levels in M2 subset. Pretreatment of M2 with resveratrol prevented the upregulation of both active p50 and p65 in response to 7-oxo-cholesterol. M1 (□) and M2 (■) macrophages pretreated or not with resveratrol (30 *μ*M) for 30 minutes were cultured for 1 hour with or without 7oxo-C (15 *μ*M). Cells were then analyzed by NF-*κ*B (p50 and p65) transcription factor assay to monitor NF-*κ*B activation. The results are expressed as arbitrary units (*n* = 3, p50: *P < 0.01; ^†^ *P* < 0.001; p65: *P < 0.05; ^†^ *P* < 0.01).

7-oxo-cholesterol treatment significantly increased active NF-*κ*B p50 and p65 levels in M2 macrophages (Figure 5). It also tended to increase the p65 levels in the M1 subset, although not in a statistically significant way. Pretreatment of macrophages with resveratrol prevented the upregulation of active p50 and p65 in response to 7-oxo-cholesterol in the M2 subset.

## 4. Discussion

In the present study we demonstrated that resveratrol, a known antioxidant and anti-inflammatory natural phenolic compound [20], possesses immunomodulatory and anti-inflammatory activities in human M1 and M2 macrophages challenged with 7-oxo-cholesterol, a cholesterol autoxidation product.

In a recent study, we demonstrated that 7-oxo-cholesterol affects human macrophage biology by switching M2 macrophages from an anti- to a proinflammatory and proatherogenic M1-like phenotype [15]. We postulated that this new pathway may have implications in atherosclerotic disease where oxidative stress, which generates oxidized lipids, and cell-based inflammatory mechanisms are tightly connected. In this same study, we demonstrated by surface markers that 7-oxo-cholesterol-stimulated M1 macrophages exhibit an increased expression of the activation marker HLA-DR, even more pronounced than that one caused by LPS [15]. This points to an upregulation of macrophage function as antigen presenting cells that favour the activation of adaptive immune responses. In the same subset, we demonstrated that 7-oxo-cholesterol is able to downregulate CD16, a low affinity Fc receptor for IgG antibodies, thus likely impairing the phagocytosis of antibody-antigen complexes [21]. In our present study, we confirmed the effects of 7-oxo-cholesterol on M1 cell phenotype and demonstrated that resveratrol was able to prevent the oxysterol-induced phenotypical changes (Figure 1). In this way, resveratrol may exert an anti-inflammatory activity by limiting the activation of the immune system and preserving the anti-inflammatory clearance capacity of M1 cells [21]. Concerning the effects of 7-oxo-cholesterol on M2 subset, we confirmed its ability to increase surface expression of the monocyte differentiation antigen CD14, a pattern recognition coreceptor for bacterial LPS and cell-activating mediator of inflammatory responses [22]. Resveratrol was able to counteract the oxysterol-induced switch of the M2 subset to a more pronounced proinflammatory phenotype (Figure 1).

Numerous investigations indicate that—beside phenotype—a main difference between different polarized macrophage subsets lies in the production of key cytokines and chemokines, proteases, and other mediators [23]. Macrophages are major components of the innate immune system. The activation of macrophages has been shown to play a pivotal role during the initiation and development of inflammatory responses by producing numerous proinflammatory mediators [24].

Macrophages are a significant source of extracellular proteases, including MMPs, as well as of pro- and anti-inflammatory cytokines that regulate extracellular matrix remodelling, inflammatory cell recruitment and activation, and vascular smooth muscle cell proliferation and apoptosis. All these events play a role in the progression of atherosclerotic lesions and facilitate an unstable phenotype [15]. To better investigate the impact of resveratrol on M1 and M2 macrophage subsets, we analyzed the macrophage endocytic activity, the release of two key metalloproteinases, and the secretome profile of several cytokines, chemokines, and growth factors in oxysterol-stimulated macrophages pretreated or not with resveratrol. Under physiological conditions, macrophages promote tissue homeostasis by clearing debris and preventing excessive inflammation in response to environmental stress [25]. This represents a hallmark function of M2-like macrophages that usually express higher levels of surface scavenger, mannose, and galactose-type receptors that are involved in debris clearance as compared to M1 cells [26]. In the present study, we confirmed the ability of 7-oxo-cholesterol to decrease the high endocytic clearance capacity of M2 macrophages [19] and demonstrated the ability of resveratrol to preserve this fundamental anti-inflammatory property of the M2 subset.

It is known that proinflammatory M1 macrophages release higher amounts of MMPs than the anti-inflammatory M2 cells. We have previously demonstrated that 7-oxo-cholesterol increases the ability of M2 cells to secrete MMP-9 [15] and in the present study we demonstrated that it also upregulates the expression of MMP-2 in M1 and M2 subsets, supporting the concept that this oxysterol is able to polarize macrophages toward a proinflammatory state. As further evidence, we here provided a relevant outcome on the inhibitory effect of resveratrol upon MMP-2 and MMP-9 activity upregulation in macrophages pretreated with resveratrol before the challenge with 7-oxo-cholesterol. Our results agree with previous findings by Walker et al. [20] who demonstrated that resveratrol is able to downregulate PMA-mediated induction of MMP-9 activity in U-937 macrophages by inhibiting MMP-9 gene transcription.

The present study clearly showed that resveratrol is able to modulate the release of many cytokines, chemokines, and growth factors in M1 and M2 macrophages in response to 7-oxo-cholesterol.

In our previous study, analysis of cytokine, chemokine, and growth factor secretion profile by means of a multiplexed bead assay system showed that 7-oxo-cholesterol selectively activated in both macrophage subsets the production of many key proatherogenic mediators involved in proinflammatory, proinvasive, and proangiogenic mechanisms within the atherosclerotic plaque [15]. We had previously observed that 7-oxo-cholesterol in M1 cells raised the production of the proinflammatory cytokines TNF-*α* and IL-6, thus leading to incremental proinflammatory attitude of these cells. Interestingly, 7-oxo-cholesterol induced M2 subset to release TNF-*α* and IL-6 and the M1-polarizing cytokine IL-12 [27], thus further confirming the ability of 7-oxo-cholesterol to skew M2 cell polarization towards an M1-like phenotype. Notably, in this present study, we investigated the anti-inflammatory effects of resveratrol on the secretion of the same panel of cytokines, chemokines, and growth factors by M1 and M2 macrophages. We found that resveratrol pretreatment significantly prevented TNF-*α* and IL-6 upregulation in response to 7-oxo-cholesterol in M1 cells and of TNF-*α* and IL-12 in M2 ones, thus confirming the ability of this compound to counteract the proinflammatory signaling of oxysterol in macrophages. The anti-inflammatory and immunomodulatory activities of resveratrol were further confirmed by the inhibition of many chemokines in both subsets, particularly IL-8, MCP-1, CCL3, CCL4, and RANTES and of the growth factors G-CSF and GM-CSF (in M1 cells) and VEGF (in M2 cells). These inflammatory mediators, beside their active role in recruiting leukocytes into inflammatory sites, may stimulate endothelial cell migration, spreading, and neo-vessel formation, thus promoting the angiogenesis associated with the progression of atherosclerotic plaque [28]. The inhibitory effects of resveratrol on TNF-*α*, IL-6, IL-8, MCP-1, CCL-4, RANTES, and G-CSF in the M1 macrophage subset and on TNF-*α*, IL-12, IL-8, MCP-1, CCL3, CCL-4, RANTES, and VEGF in M2 macrophages together with metalloproteinases inhibition may be added to a variety of resveratrol antiatherogenic actions, since these molecules are known to be involved in inflammatory responses in arterial walls during progression of atherosclerosis [29].

Our results are in accordance with the inhibitory effect of resveratrol on the release of proinflammatory mediators shown in various cell models after stimulation with lipopolysaccharides and in* in vivo* animal models [30–36]. Walker et al. [20], in U-937 cells stimulated with lipopolysaccharides from* Escherichia coli*, proved that 10 mM resveratrol completely inhibited the* E. coli*-LPS-induced release of IL-6 and reduced TNF-*α* release by 48.1%. In accordance with Walker et al. [20], Qureshi et al. [37] showed that 0.1 to 10 mM resveratrol inhibited the LPS-stimulated release of TNF-*α* and gene expression of TNF-*α*, IL-1*β*, IL-6, and iNOS from RAW 264.7 macrophages. Another study showed that pretreatment of RAW 264.7 macrophages with resveratrol (≥25 *μ*M) followed by LPS stimulation resulted in a reduction of the IL-6 and TNF-*α* release compared to the LPS treatment [30].

To investigate potential resveratrol mechanism(s) implicated in the prevention of macrophage proinflammatory activation in response to 7-oxo-cholesterol we analyzed the effects on the modulation of NF-*κ*B, the prototypical transcription factor, which plays a central role in innate immune response [38]. In our study we confirmed previous findings on the ability of oxysterols to trigger NF-*κ*B activation [39–41]. In particular we observed that 7-oxo-cholesterol enhanced nuclear binding activity of NF-*κ*B p50 and p65 in M2 macrophages and that resveratrol completely prevented such signaling pathway activation. This observed effect of resveratrol is in agreement with previous investigations showing that resveratrol is able to downregulate inflammatory responses through this mechanism [19, 42]. Resveratrol exists as two isomers, cis- and trans-resveratrol [24]. The cis isomer is thought to be produced naturally during grape fermentation as a result of isomerization of the trans isomer by yeast isomerases; in addition, cis-resveratrol can be obtained by exposure of the trans isomer to sunlight [24]. Huang et al. [24] provided findings that cis-resveratrol produces anti-inflammatory effects by inhibiting both the canonical and noncanonical inflammasomes, and associated pathways in human macrophages.

The lack of strong clinical/scientific evidence prompted scepticism in many cardiologists regarding the cardioprotective effects through interventions with specific dietary molecules or food-derived concentrates [43]. However, in a number of studies with large cohorts, cardiologists began to consider that the percentage of decrease in deaths from coronary heart disease attributed to risk factor changes through the implementation of healthy lifestyles, including the diet, could be higher than the percentage attributed to specific treatments. To date, and according to the clinical trials conducted so far in cardiovascular disease-prevention patients, resveratrol may exert cardioprotection by improving inflammatory, fibrinolytic, and atherogenic profiles, as well as improving glucose metabolism and endothelial function. However, the specific mechanisms related to these effects and the doses needed to achieve an optimum benefit/risk ratio have not been unequivocally established so far. In addition, the actual metabolite(s) responsible for the effects is not known. It has to be taken into account that chemical instability and low resveratrol preparation yields have limited its biopharmaceutical application [44]. In an effort to overcome these problems and enhance the pharmacological activity of resveratrol, several groups have attempted to synthesize and derivatize resveratrol [44].

## 5. Conclusion

Our study is, to the best of our knowledge, the first study showing effects of resveratrol on phenotype and function of human M1 and M2 macrophages. Taken together, the results presented here strengthen previous findings on the immunomodulatory effects of resveratrol on innate immune cells and highlight the role of resveratrol as potential therapeutic compound to counteract the proatherogenic oxysterol signaling in the macrophage subsets within atherosclerotic plaque.

Ultimately, although our study does not provide evidence on the resveratrol mechanisms and metabolite(s) related to the observed immunomodulatory effects, it is nevertheless evident that our* in vitro* model could be useful to screen the immunomodulatory effects of pharmacologically active resveratrol derivatives that exhibit anti-inflammatory properties with higher chemical stability and lower cytotoxicity.

In addition, it proves to be useful when investigating the interaction of resveratrol and resveratrol derivatives with other anti-inflammatory and antiatherogenic compounds.

## Figures and Tables

**Table 1:** Flow cytometric analysis of differentiation and activation surface markers on M1 and M2 macrophage subsets.

Surface markers		Ctr	LPS	7oxo-C	Resveratrol	Resveratrol + 7oxo-C	*P* value
M1							
CD14	%	98.6 ± 1.1	100.0 ± 0.0	98.0 ± 3.5	99.3 ± 1.1	98.6 ± 1.1	NS
	MFI	1810.0 ± 569.1	3107.0 ± 1128.0	2457.0 ± 737.8	1743.0 ± 568.1	1837.0 ± 304.8	NS
CD16	%	30.7 ± 14.0	39.0 ± 6.0	35.0 ± 7.0	25.0 ± 15.0	22.7 ± 12.0	NS
	MFI	1001.0 ± 107.5^∗†^	625.0 ± 52.0*	472.0 ± 148.0^†‡^	809.3 ± 121.1	977.0 ± 98.3^‡^	^∗‡^<0.05 ^†^<0.001
CD163	%	8.3 ± 0.6	35.7 ± 23.0	15.7 ± 5.8	8.7 ± 0.6	8.6 ± 0.6	NS
	MFI	1113 ± 554.1	1046.0 ± 461.4	999.7 ± 650.9	1061.0 ± 191.0	1015.0 ± 179.7	NS
HLA-DR	%	99.7 ± 0.6	98.3 ± 2.0	99.3 ± 0.6	99.3 ± 0.6	99.3 ± 0.6	NS
	MFI	1343.0 ± 239.8^∗†^	5296.0 ± 1264.0	5601.0 ± 777.8*	1632.0 ± 178.1	5883.0 ± 2427.0^†^	<0.05

M2							
CD14	%	93.3 ± 9.9	99.3 ± 1.1	98.0 ± 3.5	99.3 ± 1.1	99.3 ± 1.1	NS
	MFI	2103.0 ± 149.8^∗†^	7277.0 ± 2699.0*	8199.0 ± 1501.0^†‡§^	2103.0 ± 952.6^‡^	2424.0 ± 479.8^§^	<0.001
CD16	%	43.0 ± 17.1	39.3 ± 16.0	35.3 ± 2.5	25.0 ± 8.0	27.0 ± 5.6	NS
	MFI	434.0 ± 86.1	421.7 ± 137.6	448.3 ± 159.9	402.0 ± 71.2	445.3 ± 115.8	NS
CD163	%	92.7 ± 11.9	96.3 ± 5.5	93.3 ± 10.7	93.7 ± 8.5	94.0 ± 10.4	NS
	MFI	1663.0 ± 409.5	1058.0 ± 208.8	1083.0 ± 195.6	1685.0 ± 223.1	1416.0 ± 480.0	NS
HLA-DR	%	99.0 ± 0.0	98.7 ± 0.6	99.3 ± 0.6	100.0 ± 0.0	99.3 ± 0.6	NS
	MFI	3143.0 ± 1630.0	3019.0 ± 994.5	4247.0 ± 947.0	4636.0 ± 1235.0	4718.0 ± 1747	NS

Results are expressed as percentage of positive cells (%) and mean fluorescence intensity (MFI) (mean ± SD; *n* = 3). *P* values were calculated by one-way ANOVA with a Bonferroni *post hoc* test. 7oxo-C: 7-oxo-cholesterol; NS: no significance. CD16 MFI: ctr M1 versus ctr M2, *P* < 0.001; CD163 %: ctr M1 versus ctr M2, *P* < 0.05.

^∗†§‡^: indicate the statistical significant difference between numbers with the same symbol.
